# Supplementary material for: Comparison of the L3-23K and L5-Fiber Regions for Arming the Oncolytic Adenovirus Ad5-Delta-24-RGD with Reporter and Therapeutic Transgenes
Source: Int J Mol Sci. 2025 Apr 14;26(8):3700. doi: 10.3390/ijms26083700 (PMC12027834; doi:10.3390/ijms26083700)
Supplement: Supplementary file 1 [file ijms-26-03700-s001.zip › ijms-3549761-supplementary.pdf]

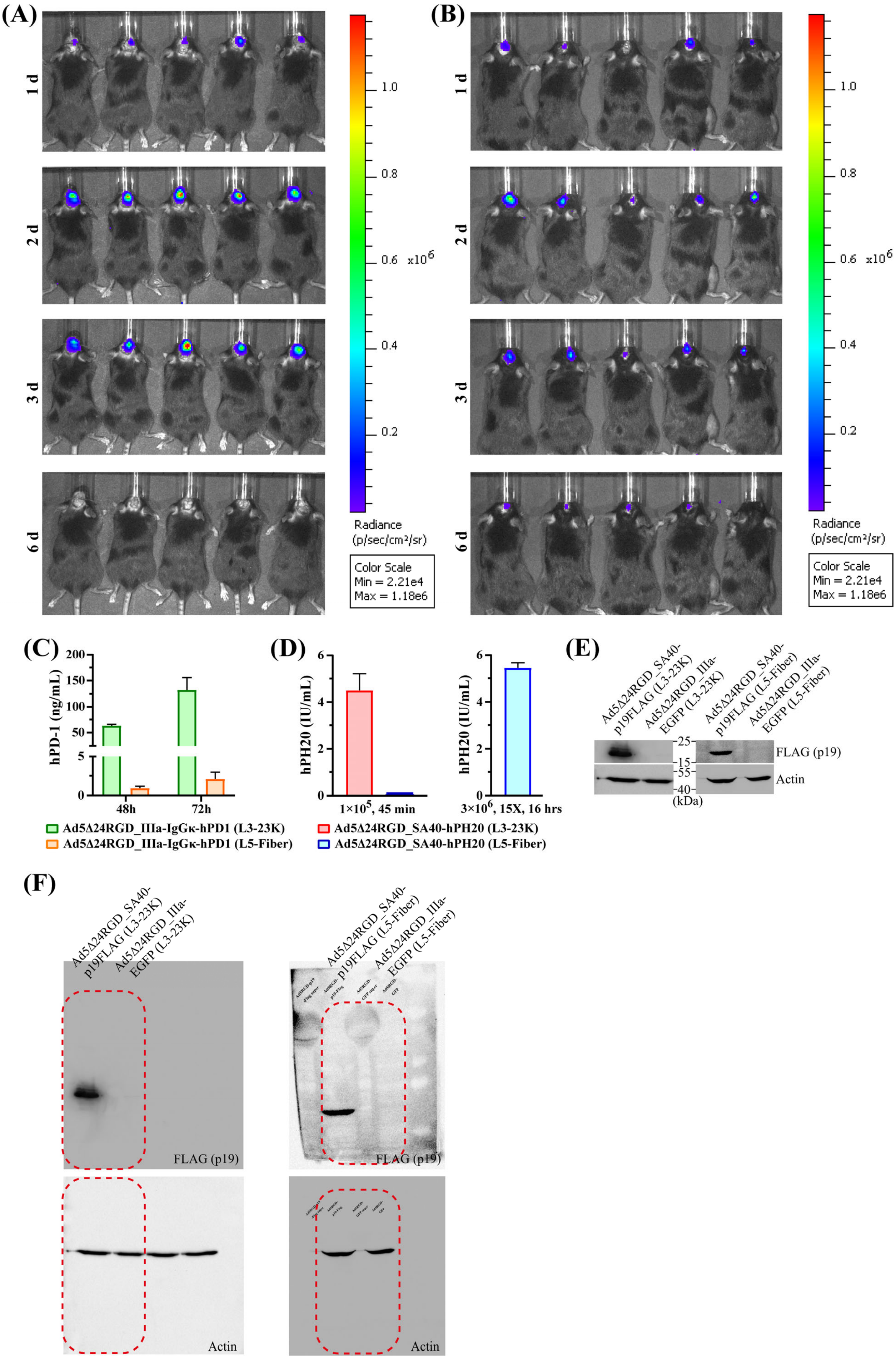

(A)
1 d
2 d
3 d
6 d
Radiance (p/sec/cm²/sr)
Color Scale Min = 2.21e4 Max = 1.18e6
(B)
(C)
hPD-1 (ng/mL)
48h
72h
Ad5Δ24RGD_IIIa-IgGκ-hPD1 (L3-23K)
Ad5Δ24RGD_IIIa-IgGκ-hPD1 (L5-Fiber)
(D)
hPH20 (IU/mL)
1×10^5, 45 min
3×10^6, 15X, 16 hrs
Ad5Δ24RGD_SA40-hPH20 (L3-23K)
Ad5Δ24RGD_SA40-hPH20 (L5-Fiber)
(E)
Ad5Δ24RGD_SA40-p19FLAG (L3-23K)
Ad5Δ24RGD_IIIa-EGFP (L3-23K)
Ad5Δ24RGD_SA40-p19FLAG (L5-Fiber)
Ad5Δ24RGD_IIIa-EGFP (L5-Fiber)
FLAG (p19)
Actin
(kDa)
(F)
FLAG (p19)
Actin

Figure S1. The production/activity of a transgene downstream of the L3-23K region is higher than that of a transgene downstream of the L5-Fiber region in human and mouse tumor cells. (**A**) Representative bioluminescence images of C57BL/6 mice (*n* = 5) at the indicated days post-inoculation of syngeneic CT-2A glioma cells (5×10^5 cells/5 μl per mouse) preinfected at an MOI of 50 IFU/cell with Ad5Δ24RGD_IIIa-Fluc (L3-23K); (**B**) Representative bioluminescence images of C57BL/6 mice (*n* = 5) with CT-2A gliomas at the indicated days post-treatment on 14 day of tumor growth with a single intratumoral injection of Ad5Δ24RGD_IIIa-Fluc (L3-23K) at a dose of 5×10^8 IFU/5 μl; (**C**) Assessment of the hPD-1 ectodomain concentrations in the supernatants of A549 cells at 48 and 72 hpi by an enzyme-linked immunosorbent assay (ELISA). Cells (1×10^5) were infected with the indicated OAds at an MOI of 5 IFU/cell. The data are shown as means (SD) of two independently repeated experiments (*N* = 2); (**D**) Determination of hyaluronidase hPH20 activity in the supernatants by a turbidimetric assay. A549 cells (1×10^5 per 48-plate well) were infected in DMEM/10% FBS with the indicated OAds at an MOI of 10 IFU/cell, and the supernatants were harvested at 48 hpi. Alternatively, A549 cells (3×10^6 per T25 flask) were infected in DMEM/1% FBS with the indicated OAds at an MOI of 20 IFU/cell. The supernatants were harvested at 48 hpi and concentrated 15-fold. Two independent measurements (*n* = 2) on different days of the same concentrated supernatants (*N* = 1 independently repeated experiment) incubated with hyaluronic acid for 45 min or 16 h were performed. The data are shown as means (SD); (**E**) Western blot analysis of p19FLAG expression with antibodies to the FLAG-tag in the lysates of A549 cells infected with the indicated OAds; (**F**) Uncropped membranes (raw data) of Western blot analysis with antibodies to the FLAG-tag and actin in the lysates of A549 cells infected with the indicated OAds.

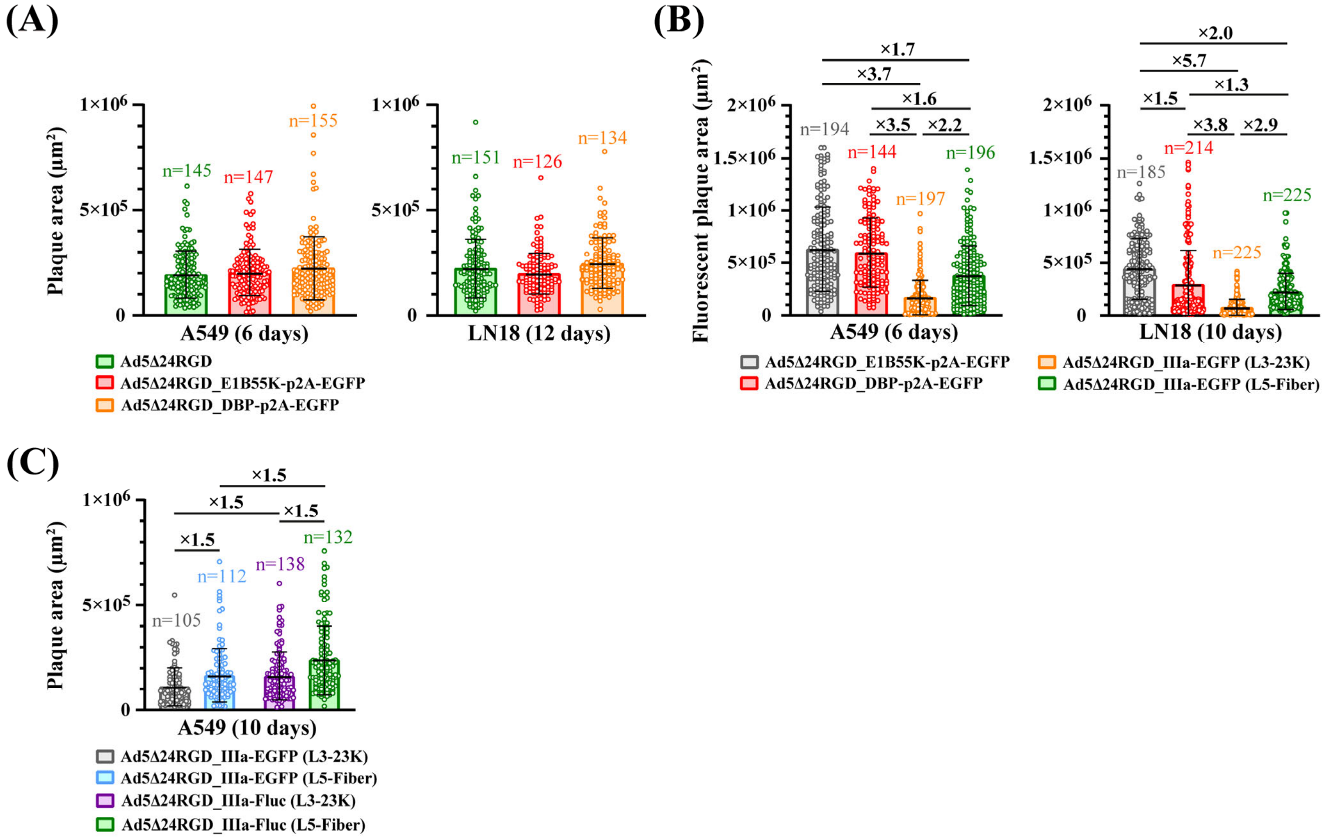

Figure S2. Insertion of EGFP and Fluc reporter transgenes downstream of the L3-23K or L5-Fiber region affects the spread and plaque formation efficacy of Ad5Δ24RGD. (**A**) Comparison of the size of MTT-stained plaques in cancer cell monolayers under the agarose overlay. The cells were seeded in 6-well plates (6×10^5 cells/well for A549 and 9×10^5 for LN18) and infected the following day with appropriate dilutions of viruses determined empirically. The sizes of 40-60 random plaques from two to three wells were taken for analysis from each independent experiment. The total number (*n*) of analyzed plaques collected from three independently repeated experiments (*N* = 3) are indicated for each group. The data are shown as means (SD) of the total number (*n*) of analyzed plaques; (**B**) Comparison of the size of fluorescent plaques in cancer cell monolayers under the agarose overlay. The cells were seeded in 6-well plates (6×10^5 cells/well for A549 and 9×10^5 for LN18) and infected the following day with appropriate dilutions of viruses determined empirically. The sizes of 30-60 random plaques from two to three wells were taken for analysis from each independent experiment. The total number (*n*) of analyzed plaques collected from four independently repeated experiments (*N* = 4) are indicated for each group. The data are shown as means (SD) of the total number (*n*) of analyzed plaques. Only ≥1.3-fold differences in the mean plaque sizes are indicated; (**C**) Comparison of the size of MTT-stained plaques in cancer cell monolayers under the agarose overlay. The cells were seeded in 6-well plates (6×10^5 cells/well for A549 and 9×10^5 for LN18) and infected the following day with appropriate dilutions of viruses determined empirically. The sizes of 30-50 random plaques from two to three wells were taken for analysis from each independent experiment. The total number (*n*) of analyzed plaques collected from three independently repeated experiments (*N* = 3) are indicated for each group. The data are shown as means (SD) of the total number (*n*) of analyzed plaques. Only ≥1.3-fold differences in the mean plaque sizes are indicated.

Insert	Length (aa)	GC3%	Total virus yield		Cytolytic activity		Plaque size	
			L3-23K	L5-Fiber	L3-23K	L5-Fiber	L3-23K	L5-Fiber
IIaSA-EGFP	239	97.1	↓	↓/≈	↓	↓	↓	↓
IIaSA-IgGκ-ecto-hPD1	142	83.1	↓	≈	≈	≈	↑/≈	↑/≈
IIaSA-Fluc	550	49.6	↓	≈	≈	↓	↓	↓
SA40-p19FLAG	180	38.3	↓	≈	≈	↑	↓/≈	≈
SA40-sPH20	489	35.8	≈	≈	≈	↑	↓/≈	↑/≈

Figure S3. Insertion of transgenes downstream of the L3-23K or L5-Fiber region differentially and unpredictably affects the oncolytic potency of Ad5Δ24RGD. The relationship between amino acid (aa) length and GC3% content of the coding sequence of transgenes and viral fitness indicators (total virus yield, cytolytic activity, and plaque size) is shown. The results were obtained from assays on A549 and LN18 cells (see Figure 3). An upward arrow (↑) indicates an increase in value, a downward arrow (↓) indicates a decrease in value, and an approximately equal sign (≈) indicates no considerable difference in value compared to the parent virus Ad5Δ24RGD.

Table S1. Nucleotide sequences inserted downstream of the L3-23K or L5-Fiber region of the Ad5Δ24RGD genome

Modification	Inserted nucleotide sequence
IIIa-EGFP (L3-23K)	agtactaagcggtgatgtttctgatcagccaccatggtgagcaagggcgaggagctgttcaccggggtggtgcccatcctggtcgagctggacggcgacgtaaacggccacaagttcagcgtgtccggcgagggcgagggcgatgccacctacggcaagctgaccctgaagttcatctgcaccaccggcaagctgcccgtgccctggcccaccctcgtgaccaccttcacctacggcgtgcagtgcttcagccgctaccccgaccacatgaagcagcacgacttcttcaagtccgccatgcccgaaggctacgtccaggagcgcaccatcttcttcaaggacgacggcaactacaagacccgcgccgaggtgaagttcgagggcgacaccctggtgaaccgcatcgagctgaagggcatcgacttcaaggaggacggcaacatcctggggcacaagctggagtacaactacaacagccacaacgtctatatcatggccgacaagcagaagaacggcatcaaggtgaacttcaagatccgccacaacatcgaggacggcagcgtgcagctcgccgaccactaccagcagaacacccccatcggcgacggccccgtgctgctgcccgacaaccactacctgagcacccagtccgccctgagcaaagaccccaacgagaagcgcgatcacatggtcctgctggagttcgtgaccgccgccgggatcactctcggcatggacgagctgtacaagtaataaaatacgaaatgaat
IIIa-Fluc (L3-23K)	agtactaagcggtgatgtttctgatcagccaccatggaagacgccaaaaacataaagaaaggcccggcgccattctatccgctggaagatggaaccgctggagagcaactgcataaggctatgaagagatacgccctggttcctggaacaattgcttttacagatgcacatatcgaggtggacatcacttacgctgagtacttcgaaatgtccgttcggttggcagaagctatgaaacgatatgggctgaatacaaatcacagaatcgtcgtatgcagtgaaaactctcttcaattctttatgccggtgttgggcgcgttatttatcggagttgcagttgcgcccgcgaacgacatttataatgaacgtgaattgctcaacagtatgggcatttcgcagcctaccgtggtgttcgtttccaaaaaggggttgcaaaaaattttgaacgtgcaaaaaaagctcccaatcatccaaaaaattattatcatggattctaaaacggattaccagggatttcagtcgatgtacacgttcgtcacatctcatctacctcccggttttaatgaatacgattttgtgccagagtccttcgatagggacaagacaattgcactgatcatgaactcctctggatctactggtctgcctaaaggtgtcgctctgcctcatagaactgcctgcgtgagattctcgcatgccagagatcctatttttggcaatcaaatcattccggatactgcgattttaagtgttgttccattccatcacggttttggaatgtttactacactcggatatttgatatgtggatttcgagtcgtcttaatgtatagatttgaagaagagctgtttctgaggagccttcaggattacaagattcaaagtgcgctgctggtgccaaccctattctccttcttcgccaaaagcactctgattgacaaatacgatttatctaatttacacgaaattgcttctggtggcgctcccctctctaaggaagtcggggaagcggttgccaagaggttccatctgccaggtatcaggcaaggatatgggctcactgagactacatcagctattctgattacacccgagggggatgataaaccgggcgcggtcggtaaagttgttccattttttgaagcgaaggttgtggatctggataccgggaaaacgctgggcgttaatcaaagaggcgaactgtgtgtgagaggtcctatgattatgtccggttatgtaaacaatccggaagcgaccaacgccttgattgacaaggatggatggctacattctggagacatagcttactgggacgaagacgaacacttcttcatcgttgaccgcctgaagtctctgattaagtacaaaggctatcaggtggctcccgctgaattggaatccatcttgctccaacaccccaacatcttcgacgcaggtgtcgcaggtcttcccgacgatgacgccggtgaacttcccgccgccgttgttgttttggagcacggaaagacgatgacggaaaaagagatcgtggattacgtcgccagtcaagtaacaaccgcgaaaaagttgcgcggaggagttgtgtttgtggacgaagtaccgaaaggtcttaccggaaaactcgacgcaagaaaaatcagagagatcctcataaaggccaagaagggcggaaagatcgccgtgtaataaaa
IIIa-IgGκ-hPD1 (L3-23K)	agtactaagcggtgatgtttctgatcagccaccatggagacagacacactcctgctatgggtactgctgctctgggttccaggttccactggtgactccccagacaggccctggaacccccccaccttctccccagccctgctcgtggtgaccgaaggggacaacgccaccttcacctgcagcttctccaacacatcggagagcttcgtgctaaactggtaccgcatgagccccagcaaccagacggacaagctggccgccttccccgaggaccgcagccagcccggccaggactgccgcttccgtgtcacacaactgcccaacgggcgtgacttccacatgagcgtggtcagggcccggcgcaatgacagcggcacctacctctgtggggccatctccctggcccccaaggcgcagatcaaagagagcctgcgggcagagctcagggtgacagagagataataaaa
IIIa-IgGκ-$hPD1_{HA}$ (L3-23K)	agtactaagcggtgatgtttctgatcagccaccatggagacagacacactcctgctatgggtactgctgctctgggttccaggttccactggtgactccccagacaggccctggaacccccccaccttctccccagccctgctcgtggtgaccgaaggggacaacgccaccttcacctgcagcttctccaacacatcggagagcttccacgtgatctggcaccgcgagagccccagcggccagacggacaccctggccgccttccccgaggaccgcagccagcccggccaggactgccgcttccgtgtcacacaactgcccaacgggcgtgacttccacatgagcgtggtcagggcccggcgcaatgacagcggcacctacgtgtgtggggtgatctccctggcccccaagattcagatcaaagagagcctgcgggcagagctcagggtgacagagagataataaaa
IIIa-IgGκ-mPD1 (L3-23K)	agtactaagcggtgatgtttctgatcagccaccatggagacagacacactcctgctatgggtactgctgctctgggttccaggttccactggtgacctagaggtccccaatgggccctggaggtccctcaccttctacccagcctggctcacagtgtcagagggagcaaatgccaccttcacctgcagcttgtccaactggtcggaggatcttatgctgaactggaaccgcctgagtcccagcaaccagactgaaaaacaggccgccttctgtaatggtttgagccaacccgtccaggatgcccgcttccagatcatacagctgcccaacaggcatgacttccacatgaacatccttgacacacggcgcaatgacagtggcatctacctctgtggggccatctccctgcaccccaaggcaaaaatcgaggagagccctggagcagagctcgtggtaacagagagataataaaa

SA40-p19FLAG (L3-23K)	ggcaggcgcaatcttcgcatttctttttccaggacgccaccatggaacgagctatacaaggaaacgacgctagggaacaagctaac<br>agtgaacgttgggatggaggatcaggaggtaccacttctcccttcaaacttcctgacgaaagtccgagttggactgagtggcggcta<br>cataacgatgagaccaattcgaatcaagataatccccttggtttcaaggaaagctggggtttcgggaaagttgtatttaagagatatct<br>cagatacgacaggacggaagcttcactgcacagagtccttggatcttggacgggagattcggttaactatgcagcatctcgatttttcg<br>gtttcgaccagatcggatgtacctatagtattcggtttcgaggagttagtatcaccgtttctggaggctctcgaactcttcagcatctctgt<br>gagatggcaattcggtctaagcaagaactgctacagcttgccccaatcgaagtggaaagtaatgtatcaagaggatgccctgaaggt<br>actgaaaccttcgaaaaagaaagcgaggactacaaagacgatgacgataaataaaa
SA40-hPH20 (L3-23K)	ggcaggcgcaatcttcgcatttctttttccaggagccaccatgggagtgctaaaattcaagcacatctttttcagaagctttgttaaatca<br>agtggagtatcccagatagttttcaccttccttctgattccatgttgcttgactctgaatttcagagcacctcctgttattccaaatgtgccttt<br>cctctgggcctggaatgccccaagtgaattttgtcttggaaaatttgatgagccactagatatgagcctcttctctttcataggaagcccc<br>cgaataaacgccaccgggcaaggtgttacaatattttatgttgatagacttggctactatccttacatagattcaatcacaggagtaact<br>gtgaatggaggaatccccagaagatttccttacaagaccatctggacaaagctaagaaagacattacattttatatgccagtagaca<br>atttgggaatggctgttattgactgggaagaatggagacccacttgggcaagaaactggaaacctaaagatgtttacaagaataggt<br>ctattgaattggttcagcaacaaaatgtacaacttagtctcacagaggccactgagaaagcaaaacaagaatttgaaaaggcaggga<br>aggatttcctggtagagactataaaattgggaaaattacttcggccaaatcacttgtggggttattatctttttccggattgttacaaccat<br>cactataagaaacccggttacaatggaagttgcttcaatgtagaaataaaaagaaatgatgatctcagctggttgtggaatgaaagca<br>ctgctctttacccatccatttatttgaacactcagcagtctcctgtagctgctacactctatgtgcgcaatcgagttcgggaagccatcaga<br>gtttccaaaatacctgatgcaaaaagtccacttccggtttttgcatatacccgcatagtttttactgatcaagttttgaaattcctttctcaag<br>atgaacttgtgtatacatttggcgaaactgttgctctgggtgcttctggaattgtaatatggggaaccctcagtataatgcgaagtatga<br>aatcttgcttgctcctagacaattacatggagactatactgaatccttacataatcaacgtcacactagcagccaaaatgtgtagccaag<br>tgctttgccaggagcaaggagtgtgtataaggaaaaactggaattcaagtgactatcttcacctcaacccagataattttgctattcaac<br>ttgagaaaggtggaaagttcacagtacgtggaaaaccgacacttgaagacctggagcaattttctgaaaattttattgcagctgttat<br>agcaccttgagttgtaaggagaaagctgatgtaaaagacactgatgctgttgatgtgtgtattgctgatggtgtctgtatagatgctttt<br>ctaaaacctcccatggagacagaagaacctcaaattttctacaatgcttcaccctccacactataataaaa
IIIa-EGFP (L5-Fiber)	agtactaagcggtgatgtttctgatcagccaccatggtgagcaagggcgaggagctgttcaccggggtggtgcccatcctggtcgag<br>ctggacggcgacgtaaacggccacaagttcagcgtgtccggcgagggcgagggcgatgccacctacggcaagctgaccctgaag<br>ttcatctgcaccaccggcaagctgcccgtgccctggcccaccctcgtgaccaccttcacctacggcgtgcagtgcttcagccgctacccc<br>gaccacatgaagcagcacgacttcttcaagtccgccatgcccgaaggctacgtccaggagcgcaccatcttcttcaaggacgacggc<br>aactacaagacccgcgccgaggtgaagttcgagggcgacaccctggtgaaccgcatcgagctgaagggcatcgacttcaaggag<br>gacggcaacatcctggggcacaagctggagtacaactacaacagccacaacgtctatatcatggccgacaagcagaagaacggca<br>tcaaggtgaacttcaagatccgccacaacatcgaggacggcagcgtgcagctcgccgaccactaccagcagaacacccccatcggc<br>gacggccccgtgctgctgcccgacaaccactacctgagcacccagtccgccctgagcaaagaccccaacgagaagcgcgatcacat<br>ggtcctgctggagttcgtgaccgccgccgggatcactctcggcatggacgagctgtacaagtaataaaatacgaaatgaat
IIIa-Fluc (L5-Fiber)	agtactaagcggtgatgtttctgatcagccaccatggaagacgccaaaaacataaagaaaggcccggcgccattctatccgctggaa<br>gatggaaccgctggagagcaactgcataaggctatgaagagatacgccctggttcctggaacaattgcttttacagatgcacatatcg<br>aggtggacatcacttacgctgagtacttcgaaatgtccgttcggttggcagaagctatgaaacgatatgggctgaatacaaatcacag<br>aatcgtcgtatgcagtgaaaactctcttcaattctttatgccggtgttgggcgcgttatttatcggagttgcagttgcgcccgcgaacgac<br>atttataatgaacgtgaattgctcaacagtatgggcatttcgcagcctaccgtggtgttcgtttccaaaaaggggttgcaaaaaattttg<br>aacgtgcaaaaaaagctcccaatcatccaaaaaattattatcatggattctaaaacggattaccagggatttcagtcgatgtacacgttc<br>gtcacatctcatctacctcccggttttaatgaatacgattttgtgccagagtccttcgatagggacaagacaattgcactgatcatgaact<br>cctctggatctactggtctgcctaaaggtgtcgctctgcctcatagaactgcctgcgtgagattctcgcatgccagagatcctatttttgg<br>caatcaaatcattccggatactgcgattttaagtgttgttccattccatcacggttttggaatgtttactacactcggatatttgatatgtgg<br>atttcgagtcgtcttaatgtatagatttgaagaagagctgtttctgaggagccttcaggattacaagattcaaagtgcgctgctggtgcc<br>aaccctattctccttcttcgccaaaagcactctgattgacaaatacgatttatctaatttacacgaaattgcttctggtggcgctcccctctct<br>aaggaagtcggggaagcggttgccaagaggttccatctgccaggtatcaggcaaggatatgggctcactgagactacatcagctat<br>tctgattacacccgagggggatgataaaccgggcgcggtcggtaaagttgttccatttttgaagcgaaggttgtggatctggatacc<br>gggaaaacgctgggcgttaatcaaagaggcgaactgtgtgtgagaggtcctatgattatgtccggttatgtaaacaatccggaagcg<br>accaacgccttgattgacaaggatggatggctacattctggagacatagcttactgggacgaagacgaacacttcttcatcgttgacc<br>gcctgaagtctctgattaagtacaaaggctatcaggtggctcccgctgaattggaatccatcttgctccaacaccccaacatcttcgacg<br>caggtgtcgcaggtcttcccgacgatgacgccggtgaacttcccgccgccgttgttgttttggagcacggaaagacgatgacggaaa<br>aagagatcgtggattacgtcgccagtcaagtaacaaccgcgaaaaagttgcgcggaggagttgtgtttgtggacgaagtaccgaaa<br>ggtcttaccggaaaactcgacgcaagaaaaatcagagagatcctcataaaggccaagaagggcggaaagatcgccgtgtaataaa<br>a

IIIa-IgGκ-hPD1 (L5-Fiber)	agtactaagcggtgatgtttctgatcagccaccatggagacagacacactcctgctatgggtactgctgctctgggttccaggttccactggtgactccccagacaggccctggaacccccccaccttctccccagccctgctcgtggtgaccgaaggggacaacgccaccttcacctgcagcttctccaacacatcggagagcttcgtgctaaactggtaccgcatgagccccagcaaccagacggacaagctggccgccttccccgaggaccgcagccagcccggccaggactgccgcttccgtgtcacacaactgcccaacgggcgtgacttccacatgagcgtggtcagggcccggcgcaatgacagcggcacctacctctgtggggccatctccctggcccccaaggcgcagatcaaagagagcctgcgggcagagctcagggtgacagagagataataaaa
SA40-p19FLAG (L5-Fiber)	ggcaggcgcaatcttcgcatttctttttccaggacgccaccatggaacgagctatacaaggaaacgacgctagggaacaagctaacagtgaacgttgggatggaggatcaggaggtaccacttctcccttcaaacttcctgacgaaagtccgagttggactgagtggcggctacataacgatgagaccaattcgaatcaagataatccccttggtttcaaggaaagctggggtttcgggaaagttgtatttaagagatatctcagatacgacaggacggaagcttcactgcacagagtccttggatcttggacgggagattcggttaactatgcagcatctcgatttttcggtttcgaccagatcggatgtacctatagtattcggtttcgaggagttagtatcaccgtttctggaggctctcgaactcttcagcatctctgtgagatggcaattcggtctaagcaagaactgctacagcttgccccaatcgaagtggaaagtaatgtatcaagaggatgccctgaaggtactgaaaccttcgaaaaagaaagcgaggactacaaagacgatgacgataaataaa
SA40-hPH20 (L5-Fiber)	ggcaggcgcaatcttcgcatttctttttccaggagccaccatgggagtgctaaaattcaagcacatctttttcagaagctttgttaaatcaagtggagtatcccagatagttttcaccttccttctgattccatgttgcttgactctgaatttcagagcacctcctgttattccaaatgtgcctttcctctgggcctggaatgccccaagtgaattttgtcttggaaaatttgatgagccactagatatgagcctcttctctttcataggaagccccgaataaacgccaccgggcaaggtgttacaatattttatgttgatagacttggctactatccttacatagattcaatcacaggagtaactgtgaatggaggaatcccccagaagatttccttacaagaccatctggacaaagctaagaaagacattacattttatatgccagtagacaatttgggaatggctgttattgactgggaagaatggagacccacttgggcaagaaactggaaacctaaagatgtttacaagaataggtctattgaattggttcagcaacaaaatgtacaacttagtctcacagaggccactgagaaagcaaaacaagaatttgaaaaggcagggaaggatttcctggtagagactataaaattgggaaaattacttcggccaaatcacttgtggggttattatctttttccggattgttacaaccatcactataagaaacccggttacaatggaagttgcttcaatgtagaaataaaaagaaatgatgatctcagctggttgtggaatgaaagcactgctctttacccatccatttatttgaacactcagcagtctcctgtagctgctacactctatgtgcgcaatcgagttcgggaagccatcagagtttccaaaatacctgatgcaaaaagtccacttccggtttttgcatatacccgcatagtttttactgatcaagttttgaaattcctttctcaagatgaacttgtgtatacatttggcgaaactgttgctctgggtgcttctggaattgtaatatggggaaccctcagtataatgcgaagtatgaaatcttgcttgctcctagacaattacatggagactatactgaatccttacataatcaacgtcacactagcagccaaaatgtgtagccaagtgctttgccaggagcaaggagtgtgtataaggaaaaactggaattcaagtgactatcttcacctcaacccagataattttgctattcaacttgagaaaggtggaaagttcacagtacgtggaaaaccgacacttgaagacctggagcaattttctgaaaaattttattgcagctgttatagcaccttgagttgtaaggagaaagctgatgtaaaagacactgatgctgttgatgtgtgtattgctgatggtgtctgtatagatgcttttctaaaacctcccatggagacagaagaacctcaaattttctacaatgcttcaccctccacactataataaa

Table S2. Oligonucleotides for homologous recombination

Name	Sequence
**rpsL-neo-E1B55K-p2A-EGFP/Fluc modification**	
Fwd_rec_rpsl-neo_E1B55K-p2A-EGFP	CTTGGTGCTGGCCTGCACCCGCGCTGAGTTTGGCTCTAGCGATGAAGATACAGATGGCCTGGTGATGATGGC
Rev_rec_rpsl-neo_E1B55K-p2A-EGFP	CACCTTATATATTCTTTCCCACCCTTAAGCCACGCCCACACATTTCAGTACCTCATCAGAAGAACTCGTCAAGAAGG
**E1B55K-p2A-EGFP modification**	
Fwd_rec w/o HA_E1B55K/DBP-p2A-EGFP	GCGGAGCTACTAACTTCAGCCTGCTGAAGCAGGCTGGTGACGTCGAGGAGAATCCTGGCCCAATGGTGAGCAAGGGCGAG
Fwd_rec_PCR-HA_E1B55K-p2A-EGFP/Fluc/hPH20	CTTGGTGCTGGCCTGCACCCGCGCTGAGTTTGGCTCTAGCGATGAAGATACAGATGGAAGCGGAGCTACTAACTTCAGCC
Rev_rec_E1B55K-p2A-EGFP	CACCTTATATATTCTTTCCCACCCTTAAGCCACGCCCACACATTTCAGTACCTCACTTGTACAGCTCGTCCATGCC
**E1B55K-p2A-Fluc modification**	
Fwd_rec w/o HA_E1B55K-p2a-FLuc	GGAAGCGGAGCTACTAACTTCAGCCTGCTGAAGCAGGCTGGTGACGTCGAGGAGAATCCTGGCCCAATGGAAGACGCCAAAAACATAAAG
Fwd_rec_PCR-HA_E1B55K-p2A-EGFP/Fluc/hPH20	CTTGGTGCTGGCCTGCACCCGCGCTGAGTTTGGCTCTAGCGATGAAGATACAGATGGAAGCGGAGCTACTAACTTCAGCC
Rev_rec_E1B55K-p2a-FLuc	TATATATTCTTTCCCACCCTTAAGCCACGCCCACACATTTCAGTACCTCACACGGCGATCTTTCCGC
**rpsL-neo-DBP-p2A-EGFP modification**	
Fwd_rpsl-neo_DBP	ACACTCTCGGGTGATTATTTACCCCCACCCTTGCCGTCTGCGCCGTTTAGGCCTGGTGATGATGGC
Rev_rpsl-neo_DBP	TGTCCCTGCCAGTGGCGCATAGCGATGCGCGGCAGAACCCCTTTGATTTTCAGAAGAACTCGTCAAGAAGG
**DBP-p2A-EGFP modification**	
Fwd_rec w/o HA_E1B55K/DBP-p2A-EGFP	GCGGAGCTACTAACTTCAGCCTGCTGAAGCAGGCTGGTGACGTCGAGGAGAATCCTGGCCCAATGGTGAGCAAGGGCGAG
Fwd_rec_PCR-HA_DBP-p2a-EGFP	TGTCCCTGCCAGTGGCGCATAGCGATGCGCGGCAGAACCCCTTTGATTTTGGAAGCGGAGCTACTAACTTC
Rev_rec_DBP-p2a-EGFP	ACACTCTCGGGTGATTATTTACCCCCACCCTTGCCGTCTGCGCCGTTTACTTGTACAGCTCGTCCATGC
**rpsL-neo-IIIa-EGFP/Fluc/p19/PD1/PH20 (L3-23K) modification**	
Fwd_rec_rpsl-neo_L3-23K	GATTAGGAGCGCCACTTCTTTTTGTCACTTGAAAAACATGTAAAAATAATGGCCTGGTGATGATGGC
Rev_rec_rpsl-neo_L3-23K	AGAGTGTACAAATAAAAGCATTTGCCTTTATTGAAAGTGTCTCTAGTACTCAGAAGAACTCGTCAAGAAGG
**IIIa-EGFP (L3-23K) modification**	
Fwd_rec_w/o HA_IIIa-EGFP (L3-23K/L5-Fib)	AGTACTAAGCGGTGATGTTTCTGATCAGCCACCATGGTGAGCAAGGG
Fwd_rec_PCR-HA_IIIa-EGFP/Fluc (L3-23K)	GATTAGGAGCGCCACTTCTTTTTGTCACTTGAAAAACATGTAAAAATAATAGTACTAAGCGGTGATGTTTCTG
Rev_rec_EGFP (L3-23K)	AGAGTGTACAAATAAAAGCATTTGCCTTTATTGAAAGTGTCTCTAGTACATTCATTTCGTATTTTATTACTTGTACAGCTC
**IIIa-Fluc (L3-23K) modification**	
Fwd_rec_w/o HA_IIIa-Fluc (L3-23K/L5-Fib)	AGTACTAAGCGGTGATGTTTCTGATCAGCCACCATGGAAGACGCCAAAAACATAAAG
Fwd_rec_PCR-HA_IIIa-EGFP/Fluc (L3-23K)	GATTAGGAGCGCCACTTCTTTTTGTCACTTGAAAAACATGTAAAAATAATAGTACTAAGCGGTGATGTTTCTG

Rev_rec_IIIa-Fluc (L3-23K)	AGAGTGTACAAATAAAAGCATTTGCCTTTATTGAAAGTGTCTCTAGT ACTTTTATTACACGGCGATCTTTCCGC
**IIIa-mPD1 (L3-23K) modification**	
Fwd_rec_w/o HA_IIIA-IgGκ-mPD1 (L3-23K)	ATCAGCCACCATGGAGACAGACACACTCCTGCTATGGGTACTGCTG CTCTGGGTTCCAGGTTCCACTGGTGACCTAGAGGTCCCCAATGGG
Fwd_rec_PCR-HA_IIIa-IgGκ-m/hPD1 (L3-23K)	GATTAGGAGCGCCACTTCTTTTTGTCACTTGAAAAACATGTAAAAAT AATAGTACTAAGCGGTGATGTTTCTGATCAGCCACCATGGAGAC
Rev_rec_mPD1(L3-23K)	AGAGTGTACAAATAAAAGCATTTGCCTTTATTGAAAGTGTCTCTAGT ACTTTTATTATCTCTCTGTTACCACGAGC
**IIIa-hPD1/hPD1$_{HA}$ (L3-23K) modification**	
Fwd_rec_w/o HA_IIIa-IgGκ-hPD1 (L3-23K)	ATCAGCCACCATGGAGACAGACACACTCCTGCTATGGGTACTGCTG CTCTGGGTTCCAGGTTCCACTGGTGACTCCCCAGACAGGCC
Fwd_rec_PCR-HA_IIIa-IgGκ-m/hPD1 (L3-23K)	GATTAGGAGCGCCACTTCTTTTTGTCACTTGAAAAACATGTAAAAAT AATAGTACTAAGCGGTGATGTTTCTGATCAGCCACCATGGAGAC
Rev_rec_hPD1 (L3-23K)	AGAGTGTACAAATAAAAGCATTTGCCTTTATTGAAAGTGTCTCTAGT ACTTTTATTATCTCTCTGTCACCCTGAGC
**40SA-PH20 (L3-23K) modification**	
Fwd_rec_40SA-hPH20 (L3-23K)	ATTAGGAGCGCCACTTCTTTTTGTCACTTGAAAAACATGTAAAAATA ATGGCAGGCGCAATCTTCGCATTTCTTTTTTCCAGGAGCCACCATGG GAGTGC
Rev_rec_hPH20 (L3-23K)	AGAGTGTACAAATAAAAGCATTTGCCTTTATTGAAAGTGTCTCTAGT ACTTTTATTATAGTGTGGAGGGTGAAGCA
**40SA-p19FLAG (L3-23K) modification**	
Fwd_rec_HA_40SA-p19FLAG (L3-23K)	GATTAGGAGCGCCACTTCTTTTTGTCACTTGAAAAACATGTAAAAAT AATGGCAGGCGCAATCTTCG
Rev_rec_p19FLAG (L3-23K)	GAGAGTGTACAAATAAAAGCATTTGCCTTTATTGAAAGTGTCTCTAG TACTTTTATTTATCGTCATCGTCTTTGTAGTCC
**rpsL-neo-IIIa-EGFP/Fluc/p19/PD1/PH20 (L5-Fib) modification**	
Fwd_rec_rpsl-neo_IIIa-EGFP (L5-Fib)	GCCACATCCTCTTACACTTTTTCATACATTGCCCAAGAATAAAGAAT CGTTTGTGTTAGGCCTGGTGATGATGGC
Rev_rec_rpsl-neo_IIIa-EGFP (L5-Fib)	TACTGAATGAAAAATGACTTGAAATTTTCTGCAATTGAAAAATAAA CACGTTGAAACATCAGAAGAACTCGTCAAGAAGG
**IIIa-EGFP (L5-Fib) modification**	
Fwd_rec_w/o HA_IIIa-EGFP (L3-23K/L5-Fib)	AGTACTAAGCGGTGATGTTTCTGATCAGCCACCATGGTGAGCAAGG G
Fwd_rec_IIIa-EGFP (L5-Fib)	CATACATTGCCCAAGAATAAAGAATCGTTTGTGTTAAGTACTAAGC GGTGATGTTTCTG
Fwd_PCR-HA_IIIa-EGFP (L5-Fib)	ATATTTGCCACATCCTCTTACACTTTTTCATACATTGCCCAAGAATAA AG
Rev_rec_EGFP (L5-Fib)	GACTTGAAATTTTCTGCAATTGAAAAATAAACACGTTGAAACAATT CATTTCGTATTTTATTACTTGTACAGCTC
**IIIa-Fluc (L5-Fib) modification**	
Fwd_rec_w/o HA_IIIa-Fluc (L5-Fib/L3-23K)	AGTACTAAGCGGTGATGTTTCTGATCAGCCACCATGGAAGACGCCA AAAACATAAAG
Fwd_rec_IIIa-EGFP/Fluc (L5-Fib)	CATACATTGCCCAAGAATAAAGAATCGTTTGTGTTAAGTACTAAGC GGTGATGTTTCTG
Fwd_PCR-HA_IIIa-EGFP/Fluc (L5-Fib)	ATATTTGCCACATCCTCTTACACTTTTTCATACATTGCCCAAGAATAA AG
Rev_rec_IIIa-Fluc (L5-Fib)	TGAATGAAAAATGACTTGAAATTTTCTGCAATTGAAAAATAAACAC GTTGAAACATTTTATTACACGGCGATCTTTCCGC
**IIIa-hPD1/hPD1$_{HA}$ (L5-Fib) modification**	

Fwd_rec_w/o HA_IIIa-IgGκ-hPD1 (L5-Fib)	ATCAGCCACCATGGAGACAGACACACTCCTGCTATGGGTACTGCTGCTCTGGGTTCCAGGTTCCACTGGTGACTCCCCAGACAGGCC
Fwd_rec_PCR-HA_IIIa-IgGκ-m/hPD1 (L5-Fib)	CCTCTTACACTTTTTCATACATTGCCCAAGAATAAAGAATCGTTTGTGTTAAGTACTAAGCGGTGATGTTTCTGATCAGCCACCATGGAGACA
Rev_rec_IIIa-hPD1 (L5-Fib)	TGAATGAAAAATGACTTGAAATTTTCTGCAATTGAAAAATAAACACGTTGAAACATTTTATTATCTCTCTGTCACCCTGAGC
**40SA-hPH20 (L5-Fib) modification**	
Fwd_rec_40SA-hPH20 (L5-Fib)	TCTTACACTTTTTCATACATTGCCCAAGAATAAAGAATCGTTTGTGTTAGGCAGGCGCAATCTTCGCATTTCTTTTTCCAGGAGCCACCATGGGAGTGC
Rev_rec_40SA-hPH20 (L5-Fib)	TGACTTGAAATTTTCTGCAATTGAAAAATAAACACGTTGAAACATTTATTATAGTGTGGAGGGTGAAGCA
**40SA-p19FLAG (L5-Fib) modification**	
Fwd_rec_40SA-p19 (Fib-L5)	TACACTTTTTCATACATTGCCCAAGAATAAAGAATCGTTTGTGTTAGGCAGGCGCAATCTTCGCATTTCTTTTTCCAGGACGCCACCATGGAACGAGC
Rev_rec_p19-FLAG (Fib-L5)	AATGACTTGAAATTTTCTGCAATTGAAAAATAAACACGTTGAAACATTTATTTATCGTCATCGTCTTTGTAGTCCTCGCTTTCTTTTTCGAAGGTTTCAG

Table S3. Oligonucleotides for cloning transgenes into a lentiviral plasmid, site-directed mutagenesis, colony screening, and sequencing

Name	Sequence
**Cloning**	
Fwd_hPD1_BamHI	AGAGGATCCGCCGCCATGCAGATCCCACAGGCG
Rev_hPD1_SalI	GTCGACTCAGAGGGGCCAAGAGCAG
Fwd_mPDI_BamHI	GAGAGGATCCGCCACCATGTGGGTCCGGCAG
Rev_mPDI_SalI	GAGAGTCGACTCAAAGAGGCCAAGAACAATGT
**Site-directed mutagenesis**	
Fwd_hPD1HA #1	CACGTGATCTGGCACCGCGAGAGCCCCAGCGGCCAGACGGACACCCTGGCCG CCTTCCCCGAG
Rev_hPD1HA #1	GGTGTCCGTCTGGCCGCTGGGGCTCTCGCGGTGCCAGATCACGTGGAAGCTCT CCGATGTGTTGGAGAAGC
Fwd_hPD1HA #2	GTGTGTGGGGTGATCTCCCTGGCCCCCAAGATTCAGATCAAAGAGAGCCTGCG
Rev_hPD1HA #2	AATCTTGGGGGCCAGGGAGATCACCCCACACACGTAGGTGCCGCTGTCATTG
**Colony screening and sequencing**	
Fwd_seq_L3-23K	GGTACCCAACTCCATGCTC
Rev_seq_L3-23K	CGGATGGTTGTGCCTGAG
Fwd_seq_L5-Fiber	ACGGTACACAGGAAACAGGA
Rev_seq_L5-Fiber	CGGGGAGAAAGGACTGTGTA

Table S4. The mean infectious unit titers per milliliter (IFU/mL) of recombinant adenoviruses (at least two independent titrations)

**№**	**Virus**	**Titer (IFU/mL)**
**1**	Ad5Δ24RGD	7×10^{11} (CsCl-purified)
**2**	Ad5Δ24RGD_IIIa-EGFP (L3-23K)	7.6×10^{11} (CsCl-purified)
**3**	Ad5Δ24RGD_IIIa-Fluc (L3-23K)	1×10^{11} (CsCl-purified)
**4**	Ad5Δ24RGD_IIIa-IgGκ-hPD1 (L3-23K)	1.9×10^{11} (CsCl-purified)
**5**	Ad5Δ24RGD_IIIa-IgGκ-hPD1$_{HA}$ (L3-23K)	3.3×10^{11} (CsCl-purified)
**6**	Ad5Δ24RGD_IIIa-IgGκ-mPD1 (L3-23K)	3.7×10^{11} (CsCl-purified)
**7**	Ad5Δ24RGD_SA40-p19FLAG (L3-23K)	7.4×10^{8} (cleared crude cell lysate);
**8**	Ad5Δ24RGD_SA40-hPH20 (L3-23K)	7.6×10^{9} (cleared crude cell lysate)
**9**	Ad5Δ24RGD_IIIa-EGFP (L5-Fiber)	3.6×10^{11} (CsCl-purified)
**10**	Ad5Δ24RGD_IIIa-Fluc (L5-Fiber)	1.9×10^{12} (CsCl-purified)
**11**	Ad5Δ24RGD_IIIa-IgGκ-hPD1 (L5-Fiber)	1×10^{10} (cleared crude cell lysate)
**12**	Ad5Δ24RGD_SA40-p19FLAG (L5-Fiber)	9.6×10^{9} (cleared crude cell lysate); 5.7×10^{11} (CsCl-purified)
**13**	Ad5Δ24RGD_SA40-hPH20 (L5-Fiber)	7.5×10^{9} (cleared crude cell lysate)
**14**	Ad5Δ24RGD_E1B55K-p2A-EGFP	6×10^{11} (CsCl-purified)
**15**	Ad5Δ24RGD_DBP-p2A-EGFP	9.8×10^{10} (CsCl-purified)
